# Investigating the Prevalence of Eating Disorders, Factors Contributing to Eating Disorders and Weight Control Methods: A Cross-Sectional Study on the Residents of Qassim Region, Saudi Arabia

**DOI:** 10.7759/cureus.71039

**Published:** 2024-10-07

**Authors:** Jolan S Alsaud, Norah Aljuaylan, Deem S Alsaloom, Anwar Alsakaker, Joud S Alfayez, Roba Alshehi

**Affiliations:** 1 Family Medicine, Qassim Health Cluster, Qassim, SAU; 2 Research and Studies Unit, Patients Friends Association in Unayzah Governorate, Qassim, SAU; 3 College of Pharmacy, Qassim University, Qassim, SAU

**Keywords:** diet, diet adherence, diet patterns, eating behavior, health behaviour

## Abstract

Background*:* Eating disorders significantly affect quality of life, body image, self-esteem, and relationships. Previous studies have mainly focused on Western populations, leaving a gap in our understanding of the cultural, social, and environmental factors in non-western populations. This cross-sectional study aimed to investigate the prevalence of eating disorders and its risk factors and examine the common methods for weight control used by affected individuals within the Qassim region of Saudi Arabia.

Materials and methods*:* Recognizing the importance of understanding the landscape of eating disorders in this population, we used diagnostic criteria from the validated Arabic version of the Diagnostic and Statistical Manual of Mental Disorders (DSM-5) to assess the severity and patterns of eating disorders. Data were collected remotely over one year using a self-administered questionnaire distributed at schools, colleges, parks, and malls. The questionnaire covered demographics, weight control methods, and diagnostic criteria. This study included 404 undiagnosed cases (n = 404) to provide a comprehensive overview of the prevalence and severity of eating disorders in this region. Statistical significance was set at p < 0.05.

Results*:* Of the participants, 12 (2.9%) reported having a diagnosed eating disorder, and the severity levels varied across different disorders. Anorexia nervosa exhibited predominantly moderate severity (264, 65.3%), with lower percentages classified as low (119, 29.5%) or high severity (21, 5.2%). Avoidant/restrictive food intake disorder demonstrated a varied distribution, with 131 (32.4%) classified as high severity, whereas bulimia nervosa was predominantly of low severity (242, 59.9%). Binge eating disorders were observed in a significant proportion of patients with moderate severity (203, 50.2%).

Conclusion*: *There is an urgent need for heightened awareness, early detection, and intervention strategies tailored to the diverse spectrum of eating disorders in this region. Furthermore, understanding common methods that affect individuals’ utilization of food and the severity and distribution of different disorders provides valuable insights into tailored interventions and support systems that promote healthier relationships between food and body image within the community.

## Introduction

Eating disorders are complex psychiatric illnesses that can profoundly affect an individual’s quality of life and social functioning and often co-occur with other psychiatric conditions such as depression, substance abuse, and anxiety disorders [1]. It encompasses a variety of conditions characterized by abnormal eating habits such as compulsive overeating, chronic undereating, self-induced vomiting, and use of dietary medications [2]. These behaviors signify underlying psychological and nutritional issues and risks to an individual’s physical and emotional well-being [3]. Neglecting bodily signals, such as hunger and fullness, can exacerbate these behaviors, contributing to body image disturbances and emotional distress [1,3].

Numerous empirical investigations have underscored the escalation in the incidence of eating disorders, both within Western societies and in certain non-Western regions. A systematic review encompassing diverse demographic parameters such as timeframes, geographical locations, gender differentials, and diagnostic criteria revealed a pronouncedly elevated prevalence of eating disorders, particularly in Western nations and among female cohorts [3]. This trend was corroborated by a study conducted in Austria that yielded analogous findings [4]. Further substantiating these observations, a study in South Australia delineated a notable surge in the prevalence of binge eating behaviors across sexes [5]. Similarly, a multicenter investigation spanning seven cities within Arab nations, including Algeria, Jordan, Kuwait, Libya, Palestine, Syria, and the United Arab Emirates, revealed a pervasive prevalence of disordered eating behaviors among adolescent populations [6]. Consistent with these findings, a cross-sectional study of medical students in Morocco showed comparable trends [7]. In the Middle Eastern context, scrutiny of the prevalence of eating disorders in Saudi Arabia revealed a marked increase, particularly among university students [8,9].

The prevalence of eating disorders in Saudi Arabian adolescents is similar to that of an adolescent in other Western countries [2,10]. The prevalence of high risk for anorexia nervosa (AN) ranged from 0.0% in Jordan to 9.5% in Oman, high risk for bulimia nervosa (BN) from 0.6% in Jordan to 1.0% in the United Arab Emirates, and high risk for binge eating disorder (BED) was 1.0% and 1.8% in Turkey and Jordan, respectively [11]. Minimal data have been published on possible risk factors for eating disorders. A study conducted in a Midwestern U.S. city noted that participants with eating disorders most frequently endorsed psychological/emotional and social problems, whereas genetics/biology and media/culture ideals were least endorsed [12]. Another study showed several biological, psychological, emotional, and social factors related to eating disorders among secondary school adolescents in Al-Basra City and a strong positive relationship between AN and students’ gender. However, there is no significant relationship between sex and other eating disorders [13]. Other studies have found that various sociocultural factors, such as media exposure, pressure for thinness, and specific personality traits, such as negative emotionality and perfectionism, are associated with an increased risk of developing eating disorders or eating disorders [14].

Many studies have supported the use of cognitive-behavioral therapy (CBT) in patients with eating disorders [15-18]. Moreover, one study explored the outcome of introducing compassion-focused therapy (CFT) into a standard treatment program for people with eating disorders and found potential benefits of using CFT with people with eating disorders [19]. Additionally, two studies suggested that Multiple Family Therapy (MFT) may benefit adolescents with eating disorders [20,21]. Furthermore, two studies have indicated that Dialectical Behavioral Therapy (DBT) appears to be effective in addressing eating disorder behaviors [22,23]. Despite the increasing awareness of eating disorders as a global health concern, there is a need for comprehensive research to elucidate the prevalence and methods used to treat these disorders in specific geographical regions. Within the context of the Qassim region in Saudi Arabia, such research assumes heightened significance, as localized data can provide valuable insights into targeted interventions and support initiatives tailored to the distinctive needs of the local population. Therefore, this study aimed to address this gap by investigating the prevalence of eating disorders and risk factors and examining the most common methods used by individuals affected in the Qassim region. By clarifying these points, the study aimed to advance the field of eating disorder research and provide valuable information for evidence-based prevention, intervention, and treatment approaches. The objective was to promote the advancement of resources, assistance, and awareness among the general population in the Qassim region by collaborating with relevant stakeholders and engaging the community.

## Materials and methods

A cross-sectional study was conducted to investigate the prevalence of eating disorders and examine the common methods used by affected individuals for weight control in the Qassim region of Saudi Arabia. This study was conducted in the general population residing in the Qassim region. Data was collected remotely through a self-administered questionnaire at any public gathering in schools, colleges, parks, malls, and boulevards. The study spanned approximately one year after receiving all necessary ethical approvals.

The Cochran formula was used to calculate the sample size required for the study, with a confidence level of 1.96, an expected prevalence of 50%, and an absolute error of 5%. Thus, the minimum required sample size was 384. A non-probability convenient sampling technique was employed. The inclusion criteria were adults aged 18 years and above residing in the Qassim region who consented to participate and had never been diagnosed with an eating disorder. Exclusion criteria encompassed individuals under 18 years of age, those residing outside the Qassim region, those known to have eating disorders, and those unable to complete the questionnaire physically or cognitively were excluded. Data were collected through a survey-based approach using a structured questionnaire designed specifically for this study. The questionnaire encompassed sections addressing general demographics, methods employed by individuals with eating disorders, and diagnostic criteria based on the Arabic Valid Diagnostic and Statistical Manual of Mental Disorders (DSM-5). Prior to the main data collection phase, a pilot study was conducted to evaluate the validity and reliability of the questionnaire. Facial validity was checked by two experienced members and one biostatistician. A small sample of 150 individuals participated in this pilot study. Their feedback was invaluable for refining the questionnaire and ensuring its effectiveness in capturing the necessary data. Based on the participants’ responses, Cronbach’s alpha was calculated for the overall questionnaire (0.752) and each domain independently. Approval was obtained from the Qassim Regional Review Board before the study commenced. After being provided an outline of the purpose of the study, confidentiality assurance, and their right to withdraw from the study at any time without repercussions, the participants provided informed consent.

Data were coded and stored in an Excel database using unique identification numbers. Access to the database for the analysis was restricted to the research team. Data collected via the questionnaire were entered into the Statistical Package for the Social Sciences (SPSS) version 26.0 (IBM Corp., Armonk, NY). Statistical analysis included calculating p-values, determining 95% confidence intervals, employing Chi-square (χ2) tests for categorical variables, and utilizing T-tests for continuous variables.

## Results

Questionnaires were administered to 512 participants. We excluded 108 participants, 96 due to incomplete information and 12 because they were suffering from eating disorders. Therefore, 404 participants were included in this study. Table 1 presents the sociodemographic characteristics and practices related to diet, eating disorders, food consumption, weight control, and body shape of the participants. This table is based on data from a sample of 416 participants. The table shows that 61.5% (n = 256) were women and 38.5% (n = 160) were men. The majority of participants fell within the age range of 18-25 years (65.1%, n = 271), with the remaining 34.9% (n = 145) aged-26-60 years. With regard to dietary practices, the majority (63.7%) did not follow special diets. However, 28.6% followed a self-prepared personal system, 4.3% followed a diet prescribed by a doctor or nutritionist, and smaller percentages followed diets advised by a sports coach (2.9%) or a psychologist (0.5%). Finally, 2.9% were reportedly diagnosed with eating disorders.

**Table 1:** Particpants' sociodemographic characteristics and practices The sociodemographic characteristics and practices related to diet, eating disorders, food consumption, weight control, and body shape among the participants (n = 416)

Characteristics		N	%
Sex	Female	256	61.5
	Male	160	38.5
Age (years)	18-25	271	65.1
	26-60	145	34.9
Follow Special diet	No, I have never done that	265	63.7
	Yes, based on a personal system that I prepared myself	119	28.6
	Yes, I follow it based on what the sports coach says	12	2.9
	Yes, prescribed by a doctor or nutritionist	18	4.3
	Yes, prescribed by a psychologist	2	.5
Diagnosed with eating disorder	No	404	97.1
	Yes	12	2.9
Food eaten (n = 404)	I eat a small amount of food	65	16.1
	I eat enough food	278	68.8
	I eat a large amount of food	61	15.1
Practices on weight control (n = 475)	Avoid eating meals as I feel a weight loss	66	13.9
	Eat regular meals of foods	177	37.3
	Exercising	48	10.1
	Eating regular meals of foods, avoiding meals as I feel a weight difference	21	4.4
	I resort to eating to relieve my sadness, anger, or anxiety about my weight and other matters	60	12.6
	Playing sports	93	19.6
	I take medications to control my weight	10	2.1
Body shape (n = 404)	Fatter than it looks	133	32.9
	My body is suitable for my weight	218	54.0
	Thinner than it looks	53	13.1

Regarding eating disorders, 2.9% (n = 12) of the participants reported being diagnosed with one. The prevalence of eating disorders and methods used to control weight in unknown cases (n = 404, 97.1%) were assessed. In terms of food consumption, most participants (n = 278, 68.8%) reported eating enough food, while 16.1% (n = 65) ate a small amount, and 15.1% (n = 61) ate a large amount. Weight control practices varied, the most common being eating regular meals (n = 177, 37.3%) and avoiding meals for weight loss (n = 66, 13.9%). A significant proportion (n = 60, 12.6%) reported resorting to eating to alleviate emotions such as sadness, anger, or anxiety. Regarding physical activity, 19.6% (n = 93) of the participants engaged in sports, and 10.1% (n = 48) reported exercising for weight control. When perceptions about body shape were assessed, approximately 54.0% (n = 218) considered their body suitable for their weight, 32.9% (n = 133) felt they were fatter than they appeared, and 13.1% (n = 53) felt thinner than they appeared.

**Figure 1:**
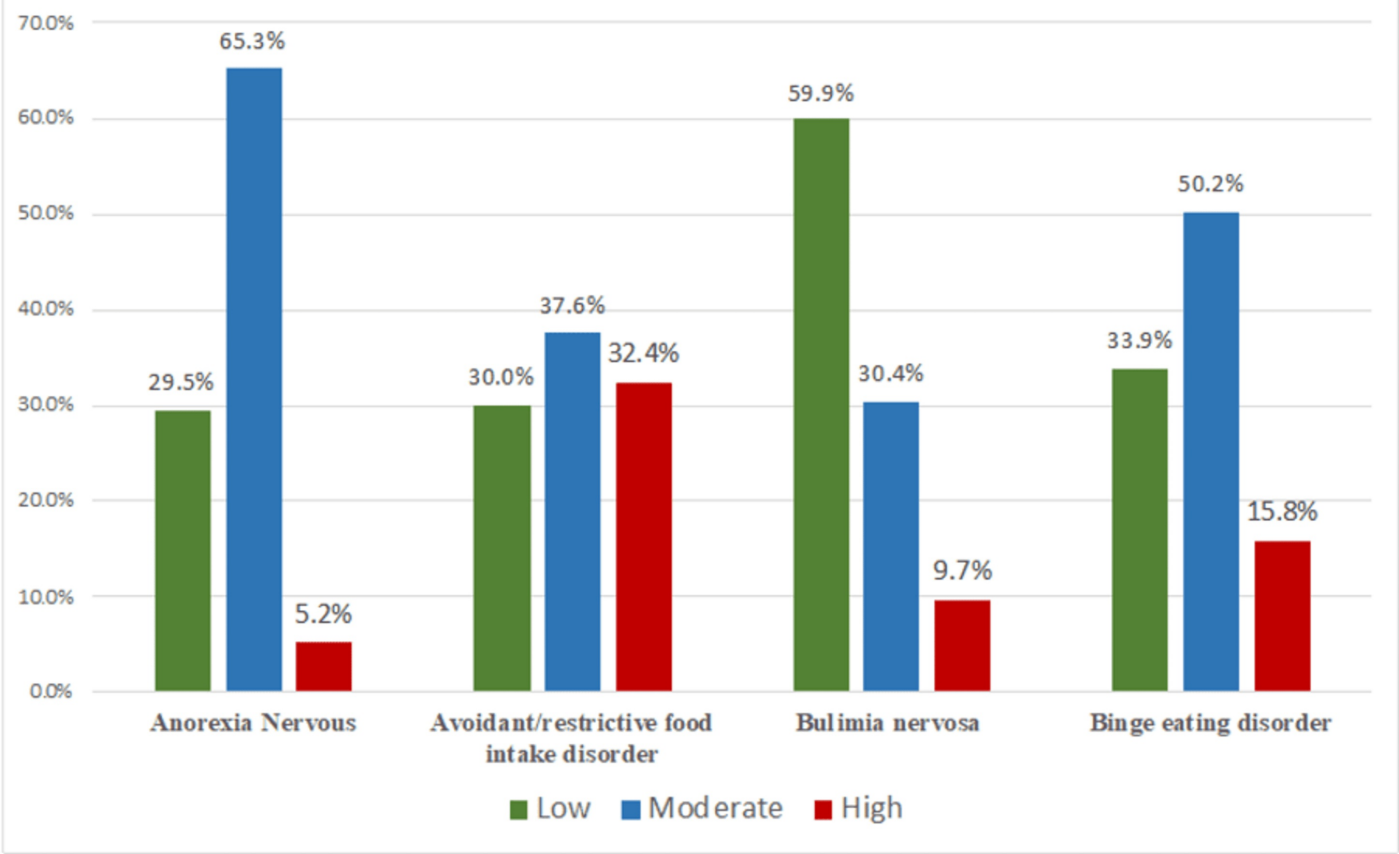
Severity of different eating disorders

The severity of eating disorders was assessed using the Eating Disorder Examination Questionnaire (EDE-Q). As shown in Table 2 and Figure 1, the distribution of severity levels varied across different eating disorders. For AN, the majority of cases (n = 264, 65.3%) were classified as moderately severe, with smaller proportions of low-(n = 119, 29.5%) and high-severity (n = 21, 5.2%) cases. In contrast, for BN, most cases (n = 242, 59.9%) were classified as low severity, followed by moderate severity (n = 123, 30.4%) and high severity (n = 39, 9.7%). The distribution of severity for avoidant/restrictive food intake disorder (ARFID) and BED was between the two patterns.

**Table 2:** Severity of the different eating disorders (n = 404)

Disorders		Severity		
		Low	Moderate	High
Anorexia Nervosa	N	119	264	21
	%	29.5	65.3	5.2
Avoidant/restrictive food intake disorder	N	121	152	131
	%	30.0	37.6	32.4
Bulimia nervosa class	N	242	123	39
	%	59.9	30.4	9.7
Binge eating disorder	N	137	203	64
	%	33.9	50.2	15.8

This variation in the severity distribution highlights the heterogeneity of eating disorders and the importance of considering individual differences when evaluating and treating these conditions. The higher prevalence of moderate- and high-severity cases in AN suggests that this disorder may require more intensive intervention than BN, where low-severity cases are more common.

Table 3 shows the relationship between eating disorders, age, and sex. Regarding the severity levels of AN, there was a trend towards the significance with age, where participants aged 26-60 had higher severity than other age groups (p = 0.069). There was a significant association with sex (p < 0.001). Moderate severity was more prevalent among males, whereas high severity was slightly more prevalent among men. For ARFID, there was a significant association with age (p = 0.007), and high severity was more prevalent among younger participants (18-25 years). However, there was no significant relationship with sex (p = 0.275). Regarding BN, there was no significant association with age (p = 0.681); however, there was a significant association with sex (p = 0.013). Higher disease severity was more prevalent among females compared to males. There was no significant association between BED and age (p = 0.145) or sex (p = 0.272), although there was a trend towards significance with sex. Overall, the severity levels appeared to be evenly distributed across age groups and sexes.

**Table 3:** Relationship between different eating with age and gender * Significant at p<0.05 level.

			Age		P value	Gender		P value
			18-25	26-60		Female	Male	
Anorexia Nervosa	Low	N	82	37	0.069	54	65	<0.001*
		%	68.9%	31.1%		45.4%	54.6%	
	Moderate	N	168	96		183	81	
		%	63.6%	36.4%		69.3%	30.7%	
	High	N	9	12		14	7	
		%	42.9%	57.1%		66.7%	33.3%	
Avoidant/restrictive food intake disorder	Low	N	69	52	0.007*	72	49	0.275
		%	57.0%	43.0%		59.5%	40.5%	
	Moderate	N	92	60		102	50	
		%	60.5%	39.5%		67.1%	32.9%	
	High	N	98	33		77	54	
		%	74.8%	25.2%		58.8%	41.2%	
Bulimia nervosa	Low	N	151	91	0.681	159	83	0.013*
		%	62.4%	37.6%		65.7%	34.3%	
	Moderate	N	82	41		76	47	
		%	66.7%	33.3%		61.8%	38.2%	
	High	N	26	13		16	23	
		%	66.7%	33.3%		41.0%	59.0%	
Binge eating disorder	Low	N	79	58	0.145	91	46	0.272
		%	57.7%	42.3%		66.4%	33.6%	
	Moderate	N	138	65		125	78	
		%	68.0%	32.0%		61.6%	38.4%	
	High	N	42	22		35	29	
		%	65.6%	34.4%		54.7%	45.3%	

The relationship between diet plans and the severity of eating disorders is shown in Table 4. For AN, there was no significant association between diet plan and severity (p = 0.460). Across all severity levels, most participants reported not following a dietary plan. Similarly, for ARFID, there was no significant relationship between diet plan and severity (p = 0.811). The distribution of severity levels was relatively consistent across the different dietary plans. In the case of BN, there was also no significant association between diet plan and severity (p = 0.320), and participants across all severity levels reported varied adherence to different diet plans. For BED, the relationship between diet plan and severity was not significant (p = 0.465), and participants reported diverse diet plan adherence regardless of the severity level.

**Table 4:** Relationship between diet plan and severity of eating disorders * Significant at p<0.05 level.

			Diet plan					
			Never	Yes, based on a personal system prepared by themself	Yes, based on what the sports coach says	Yes, prescribed by a doctor or nutritionist	Yes, prescribed by a psychologist	P value
Anorexia Nervosa	Low	N	86	26	3	3	1	0.460
		%	72.3%	21.8%	2.5%	2.5%	0.8%	
	Moderate	N	165	77	8	13	1	
		%	62.5%	29.2%	3.0%	4.9%	0.4%	
	High	N	11	8	0	2	0	
		%	52.4%	38.1%	0.0%	9.5%	0.0%	
Avoidant/ restrictive food intake disorder	Low	N	80	32	2	6	1	0.811
		%	66.1%	26.4%	1.7%	5.0%	0.8%	
	Moderate	N	97	43	3	8	1	
		%	63.8%	28.3%	2.0%	5.3%	0.7%	
	High	N	85	36	6	4	0	
		%	64.9%	27.5%	4.6%	3.1%	0.0%	
Bulimia nervosa	Low	N	156	72	5	8	1	0.320
		%	64.5%	29.8%	2.1%	3.3%	0.4%	
	Moderate	N	80	32	4	7	0	
		%	65.0%	26.0%	3.3%	5.7%	0.0%	
	High	N	26	7	2	3	1	
		%	66.7%	17.9%	5.1%	7.7%	2.6%	
Binge eating disorder	Low	N	85	41	5	6	0	0.465
		%	62.0%	29.9%	3.6%	4.4%	0.0%	
	Moderate	N	132	58	3	8	2	
		%	65.0%	28.6%	1.5%	3.9%	1.0%	
	High	N	45	12	3	4	0	
		%	70.3%	18.8%	4.7%	6.3%	0.0%	

Table 5 presents the relationship between the methods used to control weight and participants’ age and gender. Avoiding eating meals as a weight-loss method was not significantly associated with sex (p = 0.511) or age (p = 0.399). Similarly, for eating regular meals, exercising, eating to relieve emotions, and taking medications to control weight, there were no significant associations with sex or age. However, significant associations were observed for only some of these methods. Playing sports showed a significant association with sex (p = 0.026), with a higher proportion of males engaging in this method than females. Additionally, the combination of eating regular meals and avoiding meals as a weight difference was significantly associated with age (p = 0.042), with a higher proportion of younger participants adopting this method. A significant proportion of the participants reported never following a specific diet plan. This lack of awareness about healthy eating habits may contribute to the development of eating disorders. This could make it more difficult for individuals with eating disorders to seek help and recover.

**Table 5:** Relationship of methods done to control weight with age and gender of the participants * Significant at p<0.05 level.

Weight control methods			Sex		P value	Age (Years)		P value
			Female	Male		19-25	26-60	
Avoid eating meals as I feel a weight loss	No	N	213	137	0.511	231	119	0.399
		%	60.9%	39.1%		66.0%	34.0%	
	Yes	N	43	23		40	26	
		%	65.2%	34.8%		60.6%	39.4%	
Eat regular meals of foods	No	N	141	98	0.215	159	80	0.492
		%	59.0%	41.0%		66.5%	33.5%	
	Yes	N	115	62		112	65	
		%	65.0%	35.0%		63.3%	36.7%	
Exercising	No	N	225	143	0.645	235	133	0.128
		%	61.1%	38.9%		63.9%	36.1%	
	Yes	N	31	17		36	12	
		%	64.6%	35.4%		75.0%	25.0%	
Eating regular meals of foods, avoiding meals as I feel a weight difference	No	N	247	148	0.071	253	142	0.042*
		%	62.5%	37.5%		64.1%	35.9%	
	Yes	N	9	12		18	3	
		%	42.9%	57.1%		85.7%	14.3%	
I resort to eating to relieve my sadness, anger, or anxiety about my weight and other matters	No	N	217	139	0.551	231	125	0.789
		%	61.0%	39.0%		64.9%	35.1%	
	Yes	N	39	21		40	20	
		%	65.0%	35.0%		66.7%	33.3%	
Playing sports	No	N	208	115	0.026*	206	117	0.275
		%	64.4%	35.6%		63.8%	36.2%	
	Yes	N	48	45		65	28	
		%	51.6%	48.4%		69.9%	30.1%	
I take medications to control my weight	No	N	249	157	0.578	266	140	0.309
		%	61.3%	38.7%		65.5%	34.5%	
	Yes	N	7	3		5	5	
		%	70.0%	30.0%		50.0%	50.0%	

## Discussion

AN is a complex psychiatric disorder characterized by a relentless pursuit of thinness, fear of weight gain, and a distorted body image. It commonly manifests as severe dietary restrictions, excessive exercise, and other behaviors aimed at controlling weight and body shape. Most cases (264, 65.3%) were classified as moderate severity, suggesting that AN commonly presents with significant clinical features but may not always reach the extreme severity levels seen in some cases. These patients may require intensive treatment interventions, including medical monitoring, nutritional rehabilitation, and psychotherapy to address both the physical and psychological aspects of the disorder. According to diagnostic criteria outlined in the DSM-5, the annual incidence of AN in young people in the United Kingdom and Ireland is 13.68 cases per 100,000 individuals [24]. AN exhibits a predilection for onset during the peripubertal period, emphasizing its impact on individuals during this developmental stage. Epidemiological studies have demonstrated pronounced sex disparity in the incidence rate, with notably higher occurrences among women aged 15-19 years [25,26]. This demographic group constituted approximately 40% of all the identified cases, suggesting a critical period of vulnerability for the development of AN among adolescent females. Our findings also showed that the severity was significantly higher among women than men.

Numerous case-control studies have elucidated the characteristic psychometric profile of individuals susceptible to AN. Specifically, these investigations underscore the pivotal roles of perfectionism, negative affectivity, and negative self-evaluation as intrinsic factors that predispose individuals to AN development [27-30]. Of particular note are studies employing the Oxford Risk Factors Interview (RFI), which have highlighted a marked elevation in the prevalence of various personal vulnerability factors among individuals diagnosed with AN compared to control subjects [31]. These factors include perfectionism, characterized by excessively high standards and a propensity for self-criticism; negative affectivity, denoting a predisposition toward experiencing distressing emotions such as sadness, anxiety, and guilt; and negative self-evaluation, indicative of pervasive feelings of inadequacy and self-doubt [27-30,32]. High-severity AN cases, although less common in our study, represent the most critical end of the spectrum, with potentially life-threatening medical complications such as severe malnutrition, electrolyte imbalances, and cardiac issues. These individuals require immediate and comprehensive medical and psychiatric care to stabilize their condition and address the underlying psychological factors contributing to the disorder [33-35].

Unlike AN or BN, ARFID does not involve a distorted body image or a desire for weight loss. Instead, individuals with ARFID experience restrictive eating patterns often rooted in sensory sensitivity, fear of the adverse consequences of eating, or lack of interest in food [36]. The findings of this study showed that approximately 32.4% of the patients had severe ARFID, and the severity was significantly higher in younger age groups. These findings align with previous research indicating that ARFID tends to manifest at younger ages than other eating disorders such as AN [37,38]. Individuals diagnosed with ARFID frequently exhibit gastrointestinal disturbances encompassing a spectrum of symptoms ranging from discomfort to clinically significant disorders.

Moreover, this disorder is often accompanied by a constellation of medical consequences, such as inadequate nutritional intake and related physiological imbalances. Psychiatric comorbidities are prevalent among patients with ARFID, with conditions such as anxiety disorders and obsessive-compulsive disorder being frequently observed, thereby amplifying the complexity of therapeutic interventions [37-40]. The earlier onset of ARFID may have contributed to the observed higher prevalence of severe cases in younger age groups, underscoring the importance of early detection and intervention strategies tailored to address the unique needs of this population. The current study found no significant associations between the severity of any eating disorder, including ARFID, and participants' dietary plans. This lack of correlation suggests potential limitations in the sensitivity of the screening items used to detect EDs. Given that ARFID is characterized by food avoidance unrelated to weight or shape concerns, which is distinct from AN, these results imply that screening tools may not adequately capture the nuanced symptoms of ARFID. It is plausible that respondents who reported mild dietary restrictions and concerns about weight or shape without a low body mass index (BMI) may have been incorrectly categorized as positive for ARFID based solely on their endorsement of food avoidance items.

BN is characterized by an intense focus on body weight and shape, accompanied by recurrent episodes of uncontrollable overeating followed by behaviors to prevent weight gain [41]. If a person also meets the criteria for AN, the diagnosis takes precedence. BN can be challenging to detect because of the secretive behaviors of binge eating and purging. It affects approximately 0.5%-1.0% of young women, with no significant differences across social classes [3,42]. While this condition predominantly affects women, our findings showed that the severity of BN was notably higher in men than in women, with a greater proportion of women experiencing moderately severe symptoms. Unlike BN, BED involves regularly consuming abnormally large portions of food and experiencing an inability to halt eating behavior. BED is the most common eating disorder type, and approximately 3-5% of the population in the U.S. [43-45]. In a recent study conducted in Saudi Arabia, female students reported that BN and BED were the most common EDs [46]. The prevalence of BED peaks during late adolescence; however, only 11.9% of the affected adolescents actively seek clinical intervention, highlighting the critical need for early detection and preventive measures [43,44]. Moreover, binge-eating behaviors, encompassing the consumption of excessive amounts of food or experiencing a loss of control over eating, are fundamental aspects of BED. These behaviors, although more prevalent than clinically diagnosed BED, can precipitate adverse mental and physical health consequences and potentially lead to BED later in life [47]. Research has revealed a correlation between binge eating behaviors and depressive symptoms in adolescents aged 12-19 years [48]. Previous studies have reported that larger body size, exposure to negative weight-related comments, anxiety, depression, childhood trauma, food insecurity, and racism are risk factors for BED in adolescent populations [49-52]. Studies on adult populations have also reported no significant disparities in binge eating across racial or ethnic groups [53-55].

The possible limitations of this study include the use of a non-probability convenience sampling technique, which may have introduced selection bias and limited the generalizability of the findings to the broader population of the Qassim region, Saudi Arabia. Additionally, the reliance on a survey-based methodology and structured questionnaire may have introduced a response or social desirability bias, affecting the accuracy and reliability of the data collected. Furthermore, the self-reported nature of the data may have led to underreporting or overreporting of eating disorder symptoms and risk factors. Moreover, the cross-sectional design of this study limits the ability to establish causality or temporal relationships between variables. Finally, the specific cultural context of the Qassim region may introduce unique factors influencing eating behaviors and attitudes, which may not be fully captured by the study instrument.

## Conclusions

Our findings revealed that while only 12 (2.9%) of the participants reported a diagnosed eating disorder, there was a significant prevalence of undiagnosed cases. AN exhibited predominantly moderate severity, while ARFID and BED demonstrated a more varied distribution across severity levels. BN, on the other hand, predominantly manifests in low-severity cases. There is an urgent need for heightened awareness, early detection, and intervention strategies tailored to the diverse spectrum of eating disorders in this region. Furthermore, understanding the common methods that affect individuals’ utilization of food and the risk factors for disorders is crucial for developing targeted interventions and support systems to promote healthier relationships with food and body image within the Saudi population. This study provides valuable insights into the prevalence and characteristics of eating disorders in the Qassim region, paving the way for developing effective resources, assistance, and awareness programs tailored to community needs.

## References

[1] Hudson JI, Hiripi E, Pope HG Jr, Kessler RC (2007). The prevalence and correlates of eating disorders in the National Comorbidity Survey Replication. Biol Psychiatry.

[2] Jawed A, Harrison A, Dimitriou D (2020). The presentation of eating disorders in Saudi Arabia. Front Psychol.

[3] Qian J, Wu Y, Liu F (2022). An update on the prevalence of eating disorders in the general population: a systematic review and meta-analysis. Eat Weight Disord.

[4] Mangweth-Matzek B, Hoek HW, Rupp CI (2014). Prevalence of eating disorders in middle-aged women. Int J Eat Disord.

[5] Hay PJ, Mond J, Buttner P, Darby A (2008). Eating disorder behaviors are increasing: findings from two sequential community surveys in South Australia. PLoS One.

[6] Musaiger AO, Al-Mannai M, Tayyem R (2013). Risk of disordered eating attitudes among adolescents in seven Arab countries by gender and obesity: a cross-cultural study. Appetite.

[7] Attouche N, Hafdi S, Somali R, Battas O, Agoub M (2021). Factors associated with the risk of developing eating disorders among medical students in Casablanca, Morocco (Article in French). Pan Afr Med J.

[8] El-Akabawy G, Abukhaled JK, Alabdullah DW, Aleban SA, Almuqhim SA, Assiri RA (2022). Prevalence of eating disorders among Saudi female university students during the COVID-19 outbreak. J Taibah Univ Med Sci.

[9] Abd El-Azeem Taha AA, Abu-Zaid HA, El-Sayed Desouky D (2018). Eating disorders among female students of Taif University, Saudi Arabia. Arch Iran Med.

[10] Alsheweir A, Goyder E, Alnooh G, Caton SJ (2023). Prevalence of eating disorders and disordered eating behaviours amongst adolescents and young adults in Saudi Arabia: A systematic review. Nutrients.

[11] Azzeh M, Peachey G, Loney T (2022). Prevalence of high-risk disordered eating amongst adolescents and young adults in the Middle East: a scoping review. Int J Environ Res Public Health.

[12] Blodgett Salafia EH, Jones ME, Haugen EC, Schaefer MK (2015). Perceptions of the causes of eating disorders: a comparison of individuals with and without eating disorders. J Eat Disord.

[13] Baji DM, Mohammed QQ (2019). Eating disorders and its related factors among adolescents at secondary schools in Al-Basra city. Indian J Forensic Med Toxicol.

[14] Culbert KM, Racine SE, Klump KL (2015). Research review: what we have learned about the causes of eating disorders - a synthesis of sociocultural, psychological, and biological research. J Child Psychol Psychiatry.

[15] Hay P, Chinn D, Forbes D (2014). Royal Australian and New Zealand College of Psychiatrists clinical practice guidelines for the treatment of eating disorders. Aust N Z J Psychiatry.

[16] Mussell MP, Crosby RD, Crow SJ, Knopke AJ, Peterson CB, Wonderlich SA, Mitchell JE (2000). Utilization of empirically supported psychotherapy treatments for individuals with eating disorders: a survey of psychologists. Int J Eat Disord.

[17] Murphy R, Straebler S, Cooper Z, Fairburn CG (2010). Cognitive behavioral therapy for eating disorders. Psychiatr Clin North Am.

[18] Peterson CB, Mitchell JE, Engbloom S, Nugent S, Pederson Mussell M, Crow SJ, Thuras P (2001). Self-help versus therapist-led group cognitive-behavioral treatment of binge eating disorder at follow-up. Int J Eat Disord.

[19] Gale C, Gilbert P, Read N, Goss K (2014). An evaluation of the impact of introducing compassion focused therapy to a standard treatment programme for people with eating disorders. Clin Psychol Psychother.

[20] Gelin Z, Fuso S, Hendrick S, Cook-Darzens S, Simon Y (2015). The effects of a multiple family therapy on adolescents with eating disorders: an outcome study. Fam Process.

[21] Baudinet J, Eisler I, Dawson L, Simic M, Schmidt U (2021). Multi-family therapy for eating disorders: a systematic scoping review of the quantitative and qualitative findings. Int J Eat Disord.

[22] Bankoff SM, Karpel MG, Forbes HE, Pantalone DW (2012). A systematic review of dialectical behavior therapy for the treatment of eating disorders. Eat Disord.

[23] Palmer RL, Birchall H, Damani S, Gatward N, McGrain L, Parker L (2003). A dialectical behavior therapy program for people with an eating disorder and borderline personality disorder—description and outcome. Int J Eat Disord.

[24] Petkova H, Simic M, Nicholls D (2019). Incidence of anorexia nervosa in young people in the UK and Ireland: a national surveillance study. BMJ Open.

[25] Klump KL (2013). Puberty as a critical risk period for eating disorders: a review of human and animal studies. Horm Behav.

[26] Ackard DM, Peterson CB (2001). Association between puberty and disordered eating, body image, and other psychological variables. Int J Eat Disord.

[27] Fairburn CG, Cooper Z, Doll HA, Welch SL (1999). Risk factors for anorexia nervosa: three integrated case-control comparisons. Arch Gen Psychiatry.

[28] Hilbert A, Pike KM, Goldschmidt AB (2014). Risk factors across the eating disorders. Psychiatry Res.

[29] Machado BC, Gonçalves SF, Martins C (2014). Risk factors and antecedent life events in the development of anorexia nervosa: a Portuguese case-control study. Eur Eat Disord Rev.

[30] Pike KM, Hilbert A, Wilfley DE, Fairburn CG, Dohm FA, Walsh BT, Striegel-Moore R (2008). Toward an understanding of risk factors for anorexia nervosa: a case-control study. Psychol Med.

[31] Fairburn CG, Welch SL, Doll HA, Davies BA, O'Connor ME (1997). Risk factors for bulimia nervosa. A community-based case-control study. Arch Gen Psychiatry.

[32] Karwautz A, Rabe-Hesketh S, Hu X, Zhao J, Sham P, Collier DA, Treasure JL (2001). Individual-specific risk factors for anorexia nervosa: a pilot study using a discordant sister-pair design. Psychol Med.

[33] McClelland J, Hodsoll J, Brown A (2018). A pilot evaluation of a novel First Episode and Rapid Early Intervention service for Eating Disorders (FREED). Eur Eat Disord Rev.

[34] Flynn M, Austin A, Lang K (2021). Assessing the impact of First Episode Rapid Early Intervention for Eating Disorders on duration of untreated eating disorder: a multi-centre quasi-experimental study. Eur Eat Disord Rev.

[35] Frostad S, Bentz M (2022). Anorexia nervosa: outpatient treatment and medical management. World J Psychiatry.

[36] American Psychiatric Association (2013). Diagnostic and statistical manual of mental disorders (5th ed).

[37] Nicely TA, Lane-Loney S, Masciulli E (2014). Prevalence and characteristics of avoidant/restrictive food intake disorder in a cohort of young patients in day treatment for eating disorders. J Eat Disord.

[38] Norris ML, Robinson A, Obeid N (2014). Exploring avoidant/restrictive food intake disorder in eating disordered patients: A descriptive study. Int J Eat Disord.

[39] Lieberman M, Houser ME, Voyer AP, Grady S, Katzman DK (2019). Children with avoidant/restrictive food intake disorder and anorexia nervosa in a tertiary care pediatric eating disorder program: A comparative study. Int J Eat Disord.

[40] Zickgraf HF, Franklin ME, Rozin P (2016). Adult picky eaters with symptoms of avoidant/restrictive food intake disorder: comparable distress and comorbidity but different eating behaviors compared to those with disordered eating symptoms. J Eat Disord.

[41] Hay PJ, Claudino AM (2010). Bulimia nervosa. BMJ Clin Evid.

[42] AlHadi AN, Almeharish A, Bilal L, Al-Habeeb A, Al-Subaie A, Naseem MT, Altwaijri YA (2022). The prevalence and correlates of bulimia nervosa, binge-eating disorder, and anorexia nervosa: The Saudi National Mental Health Survey. Int J Eat Disord.

[43] Marzilli E, Cerniglia L, Cimino S (2018). A narrative review of binge eating disorder in adolescence: prevalence, impact, and psychological treatment strategies. Adolesc Health Med Ther.

[44] Swanson SA, Crow SJ, Le Grange D (2011). Prevalence and correlates of eating disorders in adolescents: results from the national comorbidity survey replication adolescent supplement. Arch Gen Psychiatry.

[45] Glazer KB, Sonneville KR, Micali N (2019). The course of eating disorders involving bingeing and purging among adolescent girls: prevalence, stability, and transitions. J Adolesc Health.

[46] Ali SA, Mahfouz MS, Hakami RA (2023). Prevalence and associated factors of eating disorders among female students at Jazan University, Kingdom of Saudi Arabia: a survey study. Cureus.

[47] Giel KE, Bulik CM, Fernandez-Aranda F (2022). Binge eating disorder. Nat Rev Dis Primer.

[48] Sehm M, Warschburger P (2018). Prospective associations between binge eating and psychological risk factors in adolescence. J Clin Child Adolesc Psychol.

[49] Fairburn CG, Doll HA, Welch SL (1998). Risk factors for binge eating disorder: a community-based. Case-Control Study. Arch Gen Psychiatry.

[50] Chu J, Raney JH, Ganson KT (2022). Adverse childhood experiences and binge-eating disorder in early adolescents. J Eat Disord.

[51] Raney JH, Al-Shoaibi AA, Shao IY (2023). Racial discrimination is associated with binge-eating disorder in early adolescents: a cross-sectional analysis. J Eat Disord.

[52] Nagata JM, Chu J, Cervantez L (2023). Food insecurity and binge-eating disorder in early adolescence. Int J Eat Disord.

[53] Mikhail ME, Ackerman LS, Anaya C (2023). Associations between household income and disordered eating differ across sex and racial identity in a population-based sample of adults. Int J Eat Disord.

[54] Reagan P, Hersch J (2005). Influence of race, gender, and socioeconomic status on binge eating frequency in a population-based sample. Int J Eat Disord.

[55] Simone M, Telke S, Anderson LM (2022). Ethnic/racial and gender differences in disordered eating behavior prevalence trajectories among women and men from adolescence into adulthood. Soc Sci Med.

